# Phenology and foraging bias contribute to sex‐specific foraging patterns in the rare declining butterfly *Argynnis idalia idalia*

**DOI:** 10.1002/ece3.10287

**Published:** 2023-07-18

**Authors:** Matthew W. Chmielewski, Skyler Naya, Monica Borghi, Jen Cortese, Alisdair R. Fernie, Mark T. Swartz, Konstantina Zografou, Brent J. Sewall, Rachel B. Spigler

**Affiliations:** ^1^ Department of Biology Temple University Philadelphia Pennsylvania USA; ^2^ Max Planck Institute of Molecular Plant Physiology Potsdam Germany; ^3^ The Pennsylvania Department of Military and Veterans Affairs Fort Indiantown Gap National Guard Training Center Annville Pennsylvania USA; ^4^ Present address: Department of Biology Utah State University Logan Utah USA

**Keywords:** flower visitation, intraspecific variation, long‐term ecological data, nectar chemistry, plant–pollinator interactions, sex‐specific foraging, *Speyeria idalia idalia*

## Abstract

Variation in pollinator foraging behavior can influence pollination effectiveness, community diversity, and plant–pollinator network structure. Although effects of interspecific variation have been widely documented, studies of intraspecific variation in pollinator foraging are relatively rare. Sex‐specific differences in resource use are a strong potential source of intraspecific variation, especially in species where the phenology of males and females differ. Differences may arise from encountering different flowering communities, sex‐specific traits, nutritional requirements, or a combination of these factors. We evaluated sex‐specific foraging patterns in the eastern regal fritillary butterfly (*Argynnis idalia idalia*), leveraging a 21‐year floral visitation dataset. Because *A. i. idalia* is protandrous, we determined whether foraging differences were due to divergent phenology by comparing visitation patterns between the entire season with restricted periods of male–female overlap. We quantified nectar carbohydrate and amino acid contents of the most visited plant species and compared those visited more frequently by males versus females. We demonstrate significant differences in visitation patterns between male and female *A. i. idalia* over two decades. Females visit a greater diversity of species, while dissimilarity in foraging patterns between sexes is persistent and comparable to differences between species. While differences are diminished or absent in some years during periods of male–female overlap, remaining signatures of foraging dissimilarity during implicate mechanisms other than phenology. Nectar of plants visited more by females had greater concentrations of total carbohydrates, glucose, and fructose and individual amino acids than male‐associated plants. Further work can test whether nutritional differences are a cause of visitation patterns or consequence, reflecting seasonal shifts in the nutritional landscape encountered by male and female *A. i. idalia*. We highlight the importance of considering sex‐specific foraging patterns when studying interaction networks, and in making conservation management decisions for this at‐risk butterfly and other species exhibiting strong intraspecific variation.

## Graphical abstract

Sex‐specific foraging behavior is an understudied driver of intraspecific variation in plant–pollinator relationships. We examined 21 years of flower visitation in the rare, declining butterfly *Argynnis idalia idalia* in order to determine the role that both phenology and visitation bias play in driving differences between male and female floral nectaring. Furthermore, we described variation in the concentration of floral nectar compounds in plant species differentially visited by female and males. We demonstrate that differences in sex‐specific floral visitation patterns are partially a product of both phenology and visitation bias, and that this may have implications for the nutrient landscape both sexes experience.

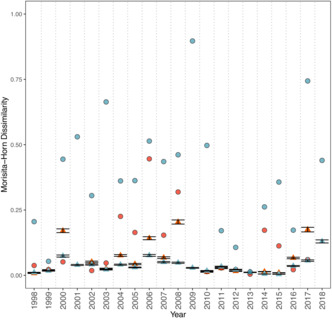

## 1 INTRODUCTION

Insect foraging behavior on flowers varies widely across species, influencing pollination success (Herrera, 1987; Spears, 1983), plant population genetic structure (Eckhart et al., 2006), and plant community composition (Valdovinos et al., 2016). In turn, the floral community affects the resources available to foragers and shapes their population and community properties (Ogilvie & Forrest, 2017). While differences in foraging behavior are typically studied between or among species, intraspecific variation in foraging occurs and may also influence pollination effectiveness (Greenleaf et al., 2007; Maruyama et al., 2016; Russell et al., 2021; Szigeti et al., 2019). Sex‐specific differences in resource use are a particularly strong potential source of reliable within‐species variation and are gaining recent attention (Kishi & Kakutani, 2020; Smith et al., 2019, 2021). Still, the frequency of sex‐specific foraging differences within pollinator species, the magnitude of any such differences, and the potential factors driving them remain poorly understood. Studies of sex‐specific foraging patterns of floral visitation present an opportunity to address these questions and, more broadly, improve our understanding of the role that intraspecific variation plays in a variety of ecological contexts from community dynamics (Bolnick et al., 2011) to floral evolution and in applied settings involving conservation decisions for at‐risk insect species.

Phenology is perhaps one of the most important ecological factors driving interspecific variation in plant–pollinator associations within plant–pollinator networks (Jordano et al., 2003; Olesen et al., 2008; Vazquez et al., 2009) and could likewise bring about male–female floral foraging differences within species. Indeed, in many butterfly and bee species, males emerge earlier than females (protandry), or in some bee species females emerge first (protogyny) (Bourke, 1997; Eickwort & Ginsberg, 1980; Roswell et al., 2019; Wiklund & Fagerström, 1977; Willmer & Stone, 2004). Emergence, peak activity, and mean flight date of males and females can diverge on the order of 2–3 weeks during the flowering season (Minckley et al., 1994; Shephard & Debinski, 2005; Zografou et al., 2021). Given flowering species turn over rapidly across the season (CaraDonna et al., 2017; Olesen et al., 2011), male and female pollinators of such species are likely to encounter divergent floral resource communities—both in terms of composition and diversity—resulting in foraging patterns that might differ as much as or even greater than differences across species. Understanding phenologically driven patterns is particularly important in the context of climate change (Visser & Both, 2005), where, for example, sex‐specific foraging could render males and females differentially vulnerable. For instance, one study found males, but not females, of a bee species emerge earlier in response to temperature (Kehrberger & Holzschuh, 2019), while another found that females, not males, of a butterfly species extended seasonal flight duration in association with climate change (Zografou et al., 2021).

A number of other mechanisms could generate sex‐specific foraging patterns, including sex‐specific sensory biases (Alarcón et al., 2010; Ogawa et al., 2013; Rutowski, 2000), differences in body size or morphology (Mendoza‐Cuenca & Macias‐Ordonez, 2005; Russell et al., 2021; Smith et al., 2019), and dietary needs (Levin et al., 2017a, 2017b). Nectar nutrition may be particularly important in defining male and female foraging patterns in butterfly species. Many rely entirely on nectar for their adult diet, and nectar nutrition can influence the fitness of both sexes (Cahenzli & Erhardt, 2012, 2013; Levin et al., 2017b; Mevi‐Schütz & Erhardt, 2005). In particular, a substantial proportion of nutrition for egg production and provisioning can be gained by females during the adult stage (Boggs & Ross, 1993; Levin et al., 2017b; Mevi‐Schütz & Erhardt, 2005; O'Brien et al., 2004). Consistent with high protein demand of egg production, studies have shown female lepidopterans may seek out nectar with greater total amino acid (AA) or specific AA content (Alm et al., 1990; Erhardt & Rusterholz, 1998; Mevi‐Schütz & Erhardt, 2003, 2004; Rusterholz & Erhardt, 2000) compared to foraging choices of males whose energetic demands are related to mate searching, though this preference is not universal (Levin et al., 2017a; Mevi‐Schütz & Erhardt, 2003, 2004, 2005). Sugars can also be important, debatably more critical for males who might need sugar‐dense nectar or simply more of it to sustain flight while patrolling and defending territories (Mendoza‐Cuenca & Macias‐Ordonez, 2005; Rusterholz & Erhardt, 2000) or for females who depend on it as a source of egg carbon (O'Brien et al., 2004, 2005). Stronger pressure for efficient foraging could also cause females to forage on fewer, high‐quality resources, whereas males may be more opportunistic and forage on a greater diversity of resources (Kishi & Kakutani, 2020; Mendoza‐Cuenca & Macias‐Ordonez, 2005; Thomas & Schultz, 2016).

General patterns of sex‐specific visitation of flower‐foraging insects are still elusive; however, with the few studies on this topic providing conflicting results (Kishi & Kakutani, 2020; Roswell et al., 2019; Rusterholz & Erhardt, 2000; Smith et al., 2021). Long‐term visitation data may be key to detecting or interpreting ecologically relevant patterns, especially if interannual fluctuations in conditions vary enough to obfuscate ecological signals during short study periods. Here, we investigated differences in foraging between males and females of the rare, declining eastern regal fritillary *Argynnis idalia idalia*, formerly *Speyeria idalia idalia* (Drury) [Lepidoptera, Nymphalidae]. First, we leveraged a 21‐year dataset of *A. i. idalia* observations to evaluate the extent to which males and females exhibit different foraging patterns. Specifically, we asked whether adult male and female *A. i. idalia* differ in floral resource use, as evidenced by the diversity and relative composition of nectar plant species visited. We then evaluated the extent to which these patterns may be driven by phenology in this protandrous species, asking whether any differences persist during periods of overlap between male and female activity. Finally, to begin probing into potential associations between nectar nutrition and sex‐specific differences in foraging behavior, we analyzed the nectar chemistry of the most visited plant species in our system, comparing those that showed greater association with either females or males.

## 2 METHODS

### 2.1 Study species and site

The regal fritillary *Argynnis idalia* is a grassland obligate species (Moranz et al., 2014) nectaring on a variety of grassland species throughout their range, including *Liatris pycnostachya*, *Monarda fistulosa*, *Echinacea pallida*, *Vernonia missurica*, *Centaurea* spp., *Cirsium* spp., *Eryngium yuccifolium*, and *Asclepias* spp. (Marschalek, 2020; Moranz et al., 2014; Zografou et al., 2017). The species once spanned longitudinally from the eastern to midwestern USA and latitudinally from Georgia, USA to Nova Scotia, Canada (Swengel, 1993). *Argynnis idalia* populations have declined as much as 95% over the past 30 years (Caven et al., 2017), and the species is under review for protection under the Endangered Species Act (USFWS, 2015). The remaining eastern range of the species is limited to two small, fragmented populations in Virginia and Pennsylvania. While the Virginia population is genetically associated with the western range, the Pennsylvania population and focus of our study has been identified as genetically distinct enough to be recommended for designation as a subspecies, *Argynnis idalia idalia*, the eastern regal fritillary (Keyghobadi et al., 2013; Williams, 2002).

**FIGURE 1:**
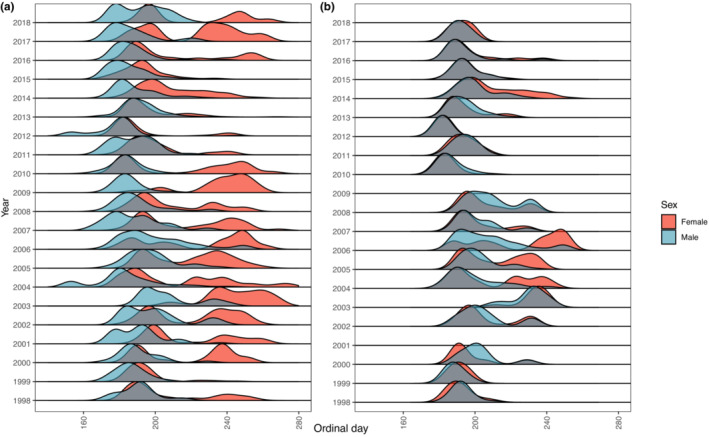
Observations of female and male floral visitations in the full (a, left) and overlap (b, right) datasets. Ordinal days are defined as day from the start of the year, with January 1 being ordinal day 1. A protandrous life history creates a scenario in which differences between male and female floral visitation differences may be partially explained by phenology. Heights and shapes of peaks represent smoothed number of visitations over time, with heights scaled within year. The overlap dataset (right) considers differences in floral visitation that occur between the first and last weeks of each year wherein females represent at least 25% but no more than 75% of observations. Overlap data for 2009 was limited to a three‐day period while 2001 and 2018 consisted of single day observation periods; these years were therefore removed from subsequent analyses involving the overlap dataset.

*Argynnis i. idalia* is protandrous, with males emerging in mid‐June and females emerging approximately 2 weeks later (Ferster & Vulinec, 2010; Zografou et al., 2017). Additionally, females are longer‐lived and undergo a reproductive diapause, which delays oogenesis thereby preventing eggs and first instar larvae desiccation during the driest part of the year. During this period, females alternate periods of rest with bouts of nectaring. This timing also aligns *A. i. idalia* larval development with leaf production in *Viola* spp., on which they feed exclusively (Kopper et al., 2001). The combination of these features of the life history of the species leads to distinct phenologies (Figure 1a), in which both sexes overlap on the landscape for only a portion of their respective adult lives (Keyghobadi et al., 2013, Williams, 2002).

This study was located within Fort Indiantown Gap National Guard Training Center (“study site”, hereafter), an active military training area in Annville, PA, USA (40°26′13.15″ N, 76°34′33.8″ W) that is home to the only *A. i. idalia* population. The study site has a humid continental climate and is in the Ridge and Valley physiographic province. The heterogeneous landscape consists of deciduous forests and temperate grasslands, with a portion of the grasslands *A. i. idalia* inhabits having been protected from military activity since 1998 (Ferster & Vulinec, 2010). These grasslands consist of primarily native flora, though some introduced species (e.g., *Centaurea* spp. known as spotted or brown knapweed) contribute to the resources available to *A. i. idalia. Argynnis i. idalia* has been monitored regularly at the study site since 1998 to inform decisions about managing remaining habitat (Keyghobadi et al., 2013).

### 2.2 Foraging observations

As part of the conservation plan for the *A. i. idalia* population at our study site, annual surveys of butterfly abundance have been conducted since 1998 across 6920 ha of grassland habitat between June and September using the Pollard Walk method (Pollard & Yates, 1994) along permanent transects (range 1557–3515 m, 10,784.06 m total) split between five designated research areas (Pennsylvania Department of Military and Veterans Affairs, 2017; Zografou et al., 2017). Surveys spanned ordinal days (e.g., where January 1 is ordinal day 1) 153–281 (June 2–October 8), encompassing the period prior to first male emergence and continuing until last female activity (Pennsylvania Department of Military and Veterans Affairs, 2017). Transects were surveyed multiple times a week in the first 3 years (1998–2000); in subsequent years, transects were surveyed weekly. Each year, 2–7 observers walked transects and recorded regal fritillary occurrences within 18.3 m (20 yards) on either side of the transect. Observers noted all occurrences of the butterfly and recorded whether male or female, based on distinct differences in color patterning on their wings. If the butterflies were nectaring, the nectar plant was identified to species and recorded. However, we note that for the two thistle species (*Cirsium pumilum* and *Cirsium discolor*, Asteraceae) some observations were recorded only as “Cirsium spp.”. We assigned these to either *C. pumilum* or *C. discolor* based on phenology via a support vector machine (see Supporting Information for details). We compiled a dataset from this long‐term survey consisting of all male and female identifications coinciding with nectaring events across a 21‐year period (1998–2018). This dataset provides information on foraging events and thus allows us to test hypotheses about differences in the frequency with which males and females forage on the observed set of nectar plants; however, without independent information on floral resource availability in the entire grassland community, we cannot directly address questions about preference, per se.

As a first step toward evaluating the extent to which sex‐specific foraging patterns occur across the season are attributable to phenology, we considered a dataset restricted to time periods when both males and females were active, which we term the “overlap” dataset (Figure 1b). We defined the period of overlap as that during which the proportion of observations was between 0.25 and 0.75 female. To determine the dates that bound this period for each year, we first tallied the number of male and female observations per week for all weeks across the 21‐year dataset. Within each year, we considered the week where females first comprised at least 25% of the observations as the starting point of the overlap period and the week where females comprised more than 75% (i.e., males dropped to below 25%) as the end point of the overlap period (Figure S1). We excluded 3 years from the overlap dataset (2001, 2009, and 2018) because our overlap criteria narrowed each to only 1–2 days of observations (Figure S1); the remaining years averaged 35 days of overlap.

### 2.3 Statistical analysis of visitation data

To assess whether male and female *A. i. idalia* differ in the number of plant species they visited, we calculated both species richness and effective species number (Jost, 2006) visited by each sex for each of the year in both our full and overlap datasets. Effective species number converts an index of information‐theoretic entropy (e.g., Shannon entropy) into an estimate of true species diversity (Jost, 2006). Whereas the nonlinear features inherent in index calculation can obscure diversity differences, effective species number allows for direct comparison of differences in the estimated number of species that two groups (e.g., sexes) interact with. For both richness and effective species number, we fit general linear mixed models including “sex” as a fixed predictor variable and “year” as a random categorical variable to account for correlations between male and female metrics within a year (richness ~ Sex + (1|Year) and effective species number ~ Sex + (1|Year)) using the nlme package ver. 3.1‐152 (Bates et al., 2015). Temporal autocorrelation in species diversity metrics could influence our results. Therefore, we applied an autocorrelation function to the model residuals, testing for lag structure via a Durbin–Watson test using car package ver. 3.0.11 wherein “year” is treated as a continuous variable. We found no significant autocorrelation at time lags of 1–4 years for effective species number (*p* > .05; Figure S2) and no significant autocorrelation at any time lag for richness (*p* > .05). While we found a significant lag 5 (5 year) autocorrelation structure for effective species number, this result was driven by a single year (2009); when removed there is no detectable autocorrelation structure out to 15 years (Figure S2).

To determine whether males and females differ in their foraging patterns, we calculated the Morisita–Horn (*d*_mh_) dissimilarity index (Horn, 1966; Morisita, 1959) between male‐ and female‐associated plant assemblages for each year and compared these via a permutation test to null distributions generated via a Patefield swap algorithm (Patefield, 1981) using the bipartite package ver. 2.16 in R (Dormann et al., 2008). This index and null model approach has been demonstrated to be reliable in other studies comparing sex‐specific differences in bipartite networks (Roswell et al., 2019; Smith et al., 2021). The Morisita–Horn index compares the probability of drawing a different species from two communities being compared with the probability of drawing from each community individually, thereby describing the difference between two communities relative to the diversity and abundance of species found in each community. The index ranges from 0 to 1, with a value of 0 interpreted as perfect similarity and 1 being interpreted as perfect dissimilarity. The Patefield swap preserves marginal totals, keeping the number of female and male butterflies and plant species the same while randomizing plant‐sex links. Observed dissimilarity values outside of the 95% CI of null distributions are then interpreted as being more (when above the upper 95% CI limit) or less (when below the lower 95% CI limit) dissimilar than a set of random associations. We considered the potential role of interannual correlation in dissimilarity both as a linear trend of *d*_mh_ over time via regression and application of the autocorrelation function using the stats package ver 4.1.0, and a Durbin–Watson test using the car package ver. 3.0.11, and found neither a linear trend (*F*_1,21_ = 0.006, *p* = .94) nor a significant autocorrelation (*p* > .05 through lag 5).

While the Morisita–Horn index provides information on female–male dissimilarity in foraging patterns, it does not specifically identify the plant species that contribute to visitation differences. To identify the plant species most influential in driving differences between male and female resource use, we conducted a Pearson's Chi‐squared analysis of floral visitation using the stats package ver. 4.1.0. To ensure robust sample sizes required for Chi‐squared analysis wherein each cell represents a plant species‐*Argynnis* sex combination, we pooled data across years in a Chi‐squared table.

We considered the extent to which phenology might account for observed differences in male and female foraging patterns and plant associations by repeating the Morisita–Horn and Chi‐squared analyses described above on the overlap dataset and comparing these with results from the full dataset. If phenology alone is associated with sex‐specific foraging patterns, we expect that differences found between males and females in the full dataset, that is, considering the entire season, will not be detectable when only considering the overlap period. The persistence of differences between the sexes in the overlap period, however, suggests the role of other mechanisms. In addition, to identify the plant species driving any remaining differences between male and female foraging patterns, we conducted two additional Chi‐squared analyses as above, using data pooled across years for the entire overlap period, and for the overlap period in only those years in which we detected a significant dissimilarity between male and female floral visitation patterns. We note that this latter step was not needed for the full dataset, since 20 of 21 years had a significantly positive *d*_mh_ index, and removing the single insignificant year yields an identical result.

All data analyses were conducted using the R statistical computing environment version 4.1.0 (R Core Team, 2021) and visualizations produced using ggplot2 ver. 3.3.5 (Wickham, 2016).

### 2.4 Nectar collection and analysis

To describe the nectar properties of the plant species most visited by *A. i. idalia*, we collected nectar from the six most visited species (*Asclepias syriaca*, *Asclepias tuberosa*, *Centaurea stoebe*, *Cirsium discolor*, *Cirsium pumilum*, *and Monarda fistulosa*) during the 2018 and 2019 flowering seasons using capillary microtubes (0.25–0.5 μL, depending on the species). Samples were collected opportunistically, with individual species being sampled over 3–7 days depending on floral availability. To prevent insect visitation prior to nectar collection, we selected newly open flowers on 8–24 plants per species and bagged them overnight with small mesh. We collected nectar from individual plants the following day between 9:00 a.m. and 02:00 p.m., pooling multiple floral units if necessary to generate biological replicates consisting of individual plants. We conducted metabolite analysis on 13 biological replicates per species (i.e., nectar from 13 different plants per species) except for *Cirsium discolor*, for which only eight replicates were used due to limited nectar availability. We extracted 2 μL of nectar from each sample, which were separated via GC–MS, annotated, integrated, and normalized by internal standard as described in Lisec et al. (2006) and Alseekh et al. (2021).

### 2.5 Statistical analysis of nectar data

We evaluated differences in total carbohydrates and total AAs as well as specific metabolites, although we provide complete metabolite data in the supplemental information (Table S1). Specifically, we examined the monosaccharides glucose and fructose and disaccharides sucrose and maltose, given their importance and abundance in nectar and prior evidence of sex‐specific preference and/or electrophysiological responses in other lepidopteran species (Alm et al., 1990; Erhardt, 1992; Romeis & Wäckers, 2000; Zhang et al., 2010). We also tested for differences in the individual AAs leucine, glycine, and proline because of evidence showing preference, responses, and fitness effects in nectaring insects (Levin et al., 2017a; Nepi et al., 2012; Terrab et al., 2007; Zhang et al., 2010). While phenylalanine has also been shown to be important, we were unable to detect it in 60% of our samples and therefore excluded it. We specifically predicted female‐associated nectars would have greater AA content given eggs are protein demanding, nectar AA can be allocated to eggs (Levin et al., 2017a, 2017b), and several studies have shown females of lepidopteran species prefer AA‐rich nectar with males indifferent or even averse (Alm et al., 1990; Erhardt, 1992; Mevi‐Schütz & Erhardt, 2002, 2003).

To test these nutrient differences in nectar of male‐ versus female‐ associated plants, we first determined whether each of the six species was male‐ or female‐associated (via Pearson's Chi‐squared analysis, see *Statistical Analysis of Visitation Data* above) and then compared nectar concentrations of total AAs, total carbohydrates, specific individual carbohydrates and selected AAs between male‐ and female‐associated plants using general linear mixed models. In each model, species identity was included as the fixed predictor variable since they were specifically chosen. However, there was significant heterogeneity of variances among species for nearly all dependent variables; we therefore also included species identity as a random group factor, which estimates variance separately for each group and adjusts all tests for unequal variances (proc glimmix in SAS software, Copyright © 2022 SAS Institute Inc). Then, we used planned contrasts that compare the difference between the averages of male‐ and female‐associated species. We note that although we analyzed and present results for both glucose and fructose, these compounds are highly correlated (*r* > .9, Figure S3). To account for multiple comparisons between compounds, we used Bonferroni‐adjusted significance values for carbohydrates (Bonferonni corrected *p*‐value = .05/5 = .01) and AAs (*p* = .05/4 = .0125).

Prior to analysis, we examined individual compound concentration data to detect and remove outliers. We defined these as individual concentration values that exceeded four times the standard deviation from the mean. This excluded a single individual *Centaurea stoebe* sample from our analysis of both proline and leucine, and a single individual sample of *Cirsium pumilum* from our analysis of glycine.

## 3 RESULTS

### 3.1 Sex‐specific foraging patterns

Across the 21 years, 6486 observations of *A. i. idalia* butterflies feeding were recorded (3264 male, 3222 female) on 19 plant species. *Asclepias tuberosa* was by far the most visited plant species, followed by *Cirsium discolor*, *Monarda fistulosa*, *Cirsium pumilum*, *Centaurea* spp., and *Asclepias syriaca* (Figure S4). Together, visits to these species represented 97.8% of all observations.

**FIGURE 2:**
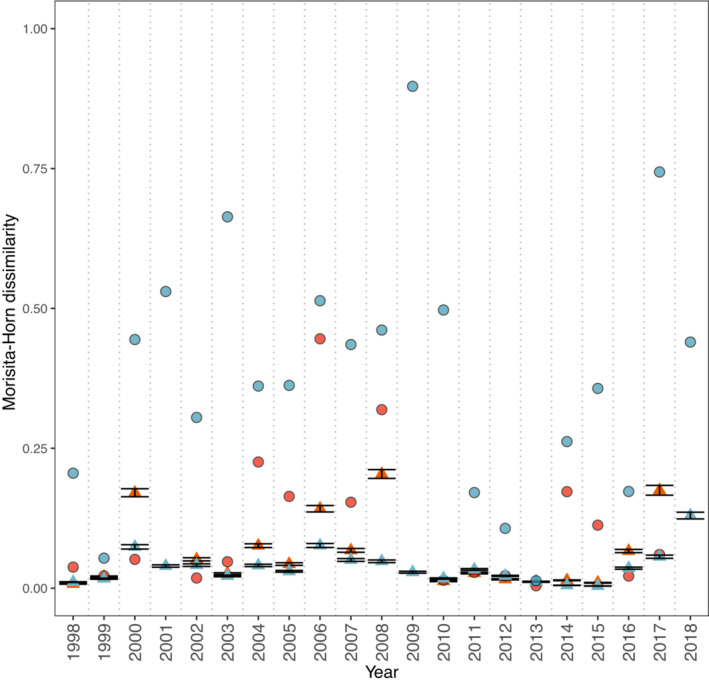
Dissimilarity of female and male regal fritillary foraging patterns as measured by the Morisita‐Horn dissimilarity index across a 21‐year period. The Morisita–Horn dissimilarity index ranges between 0 and 1; the greater the difference between male‐ and female‐associated plant assemblages, the larger the index value. Observed values (circles) were compared with a distribution of 1000 null model values (triangles) ±95% CI generated by randomizing associations in the community. Blue symbols represent the observed and expected values for the full dataset; orange‐red symbols represent observed and expected values for the overlap dataset. In the full dataset, male‐ and female‐ associated assemblages were more dissimilar than expected by chance in 20 of the 21 years studied. When phenological overlap was considered, overall dissimilarity decreased but remained greater than expected by chance in 9 of 18 years.

Across the entire flight period of males and females, species richness of nectar plants did not differ between females and males (mean 5.57 ± 0.41 SE vs. mean 5.68 ± 0.38 SE, respectively; *t*_2,38_ = 0.21, *p* = .84). However, females visited a significantly greater diversity of nectar plants on average across our sampling period as measured by effective species number (female mean 3.36 ± 0.20 SE vs. male mean 2.59 ± 0.14 SE, *t*_2,38_ = −3.27, *p* = .004). The significant difference in effective species number was not driven by any single year; effective species number of nectar plants was larger for females in 15 of 19 years. On average, the difference equates to females nectaring on 0.77 more plants than males, but within years the difference was as many as 3.14 species. Male and female *A. i. idalia* consistently differed in the relative frequency of floral species they visited across time. Specifically, the Morisita–Horn dissimilarity index was significantly greater than that expected if males and females visited floral resources randomly in 20 of 21 years (all but the year 2013; Figure 2). Mean Morisita–Horn dissimilarity across years was 0.37 (±0.05 SE).

During the overlap period, male and female butterflies did not differ in plant partner richness (female mean 4.47 ± 0.40 SE vs. male mean 3.95 ± 0.28 SE, *t*_2,38_ = −1.17, *p* = .26) or effective species number (female mean 3.04 ± 0.20 SE vs. male mean 2.66 ± 0.20 SE, *t*_2,38_ = −1.74, *p* = .10) on average. Moreover, differences in visitation patterns between sexes, measured by the *d*_mh_ index, are diminished (mean *d*_mh_ = 0.13 ± 0.04 SE, mean Δ*d*_mh_ = −0.24 ± 0.04 SE relative to the full dataset), but still greater than that expected by chance in 9 of 18 years. Patterns are indistinguishable from random in five of the years, and male and female foraging patterns were more similar than expected in four of the years (Figure 2).

**FIGURE 3:**
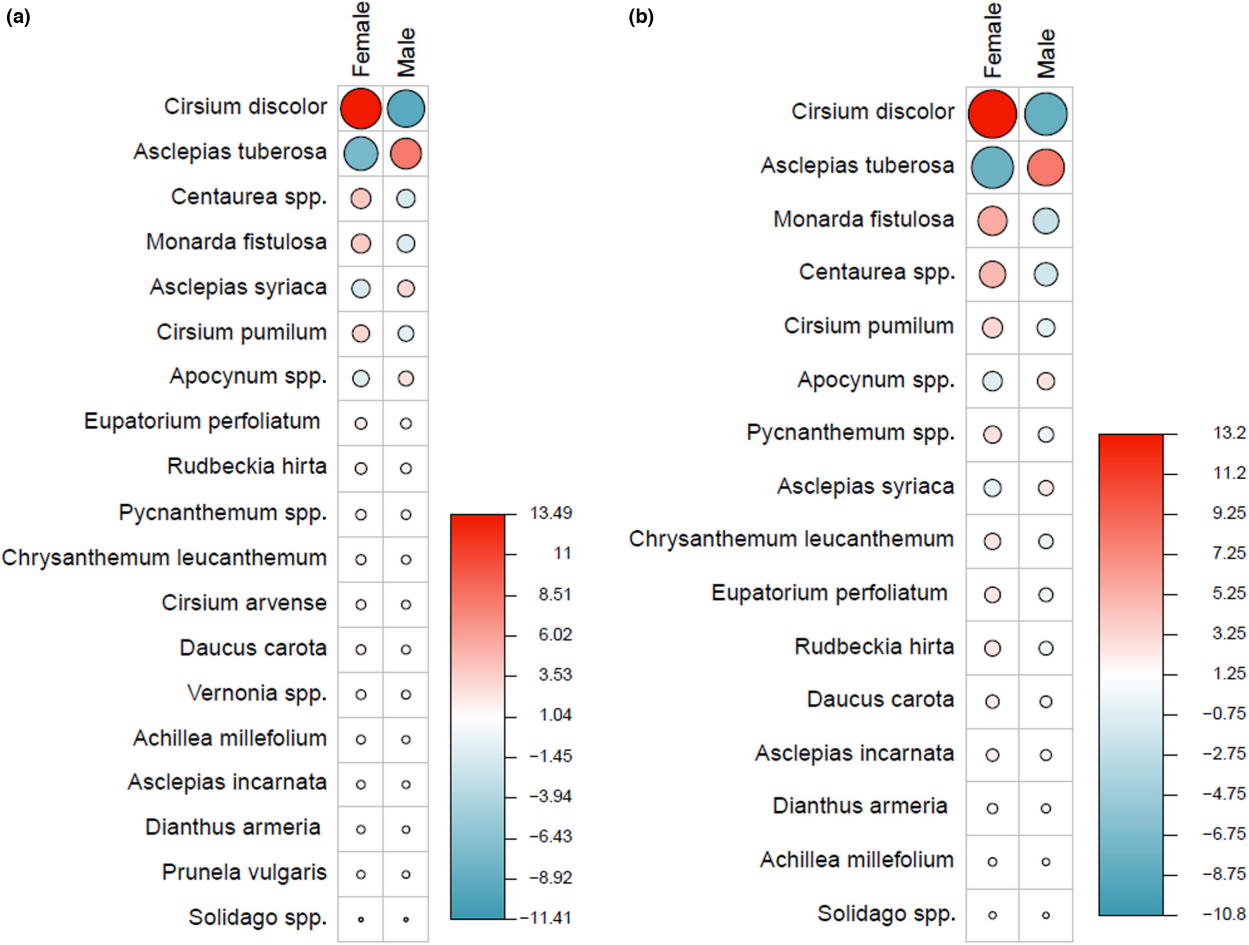
Sex‐specific associations with plant species visited by eastern regal fritillaries across a span of (a) 21 years in the full dataset comprising the entire season and (b) a restricted period of overlap between male–female flight activity, considering only those 9 years in which we detected a greater Morisita–Horn dissimilarity in the overlap data. Significant differences in visitation bias exist across all years in both datasets. The size of circles and color saturation (scale bars) indicate larger Pearson's Chi‐squared residuals and thus a greater magnitude of deviation from expected values, with red denoting a positive association (i.e., visitation is greater than that expected based on the number of times a foraging event was observed in relation to observations of males and females) and blue denoting negative association. Female and male residuals for each plant species, though similar, are not mirror images; they are a product of both row and column marginal totals and thus can vary independently.

We found that males disproportionately visited *Asclepias syriaca* and, to a lesser extent, *A. tuberosa*¸ whereas *Cirsium discolor*, *Cirsium pumilum*, *Monarda fistulosa*, and *Centaurea* spp. were visited more than expected by females (*χ* ^2^ = 788.66, *p* < .01; Figure 3a) when considering entire seasons. These differences remain robust and similar in pattern to the full dataset when considering periods of substantial female–male overlap in activity during years in which we found a significant Morisita–Horn dissimilarity index in the overlap dataset (*χ* ^2^ = 87.49, *p* < .01, Figure 3b). When considering the entire overlap dataset, the magnitude of these associations diminished (i.e., residuals from the chi‐square decreased) but remained significant (*χ* ^2^ = 83.01, *p* < .01; Figure S5). In particular, *Asclepias syriaca* and *Cirsium pumilum* became less important in driving differences between males and females in this time period.

### 3.2 Differences in floral nectar chemistry

**FIGURE 4:**
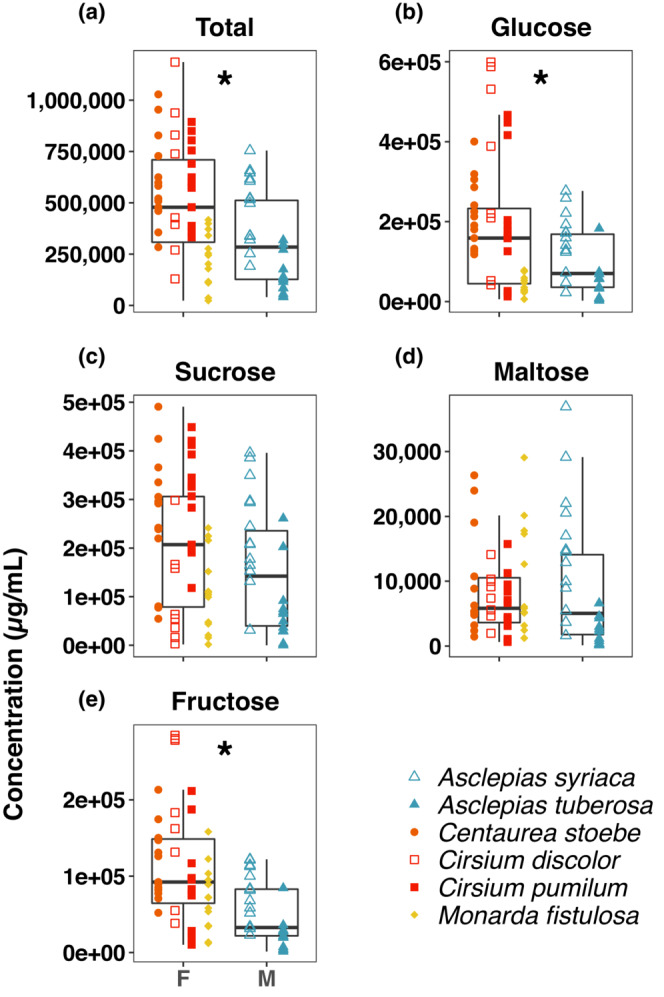
Comparisons of nectar carbohydrate concentrations for female (F)‐ versus male (M)‐associated nectar plants. We tested for differences in nectar concentrations of (a) total carbohydrates, (b) glucose, (c) sucrose, (d) maltose, and (e) fructose. Significant differences between male‐ and female‐associated species (*p* < .0125) are indicated by an asterisk. Data points represent individuals of the six plant species that contributed most to differences between female and male nectar resource use. Open versus filled shapes of the same color indicate congeneric species.

**FIGURE 5:**
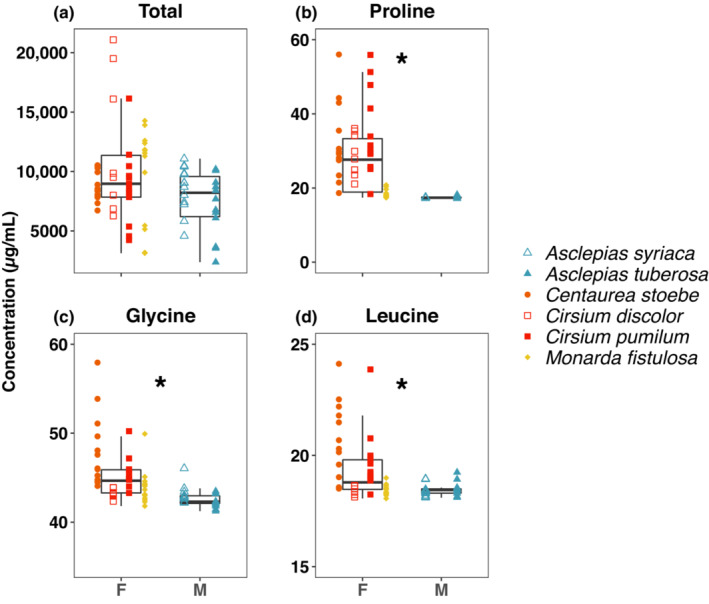
Comparisons of nectar amino acid concentrations for female (F)‐ versus male (M)‐associated nectar plants. We tested for differences in nectar concentrations of (a) total amino acids, (b) proline, (c) glycine, and (d) leucine. Significant differences between male‐ and female‐associated species (*p* < .01) are indicated by an asterisk. Data points represent individuals of the six plant species that contributed most to differences between female and male nectar resource use. Open versus filled shapes of the same color indicate congeneric species.

We were able to detect differences in foraging patterns on the six species most commonly visited and that could be considered either “male‐associated” or “female‐associated” for purposes of contrasting their nectar chemistry. *Asclepias syriaca* and *A. tuberosa* were designated as male‐associated, while *Centaurea* spp., *Cirsium discolor*, *Cirsium pumilum*, and *Monarda fistulosa* were designated as female‐associated (Figure 3). Based on this categorization, male‐ and female‐associated plants exhibited distinct nectar chemistries (see Table S1 for full nectar compound profile). Compared to the two male‐associated plant species, female‐associated plants contained significantly greater total carbohydrate content (*F*_1,62_ = 17.98, *p* < .0001) and great content of the individual carbohydrates glucose (*F*_1,62_ = 11.91, *p* < .001) and fructose (*F*_1,62_ = 27.65, *p* < .0001) (Figure 4). They also tended to have greater sucrose concentrations (*F*_1,62_ = 3.93, *p* = .05), but the difference was not significant when accounting for Bonferroni correction. In contrast, we did not detect significant differences in the level of maltose (*F*_1,62_ = 0.01, *p* = .93) (Figure 4). Total nectar AA content was similar between male‐ and female‐associated plants (*F*_1,62_ = 3.02, *p* = .08), but female‐associated plants contained significantly greater levels of the individual AAs proline (*F*_1,62_ = 78.87, *p* < .0001), glycine (*F*_1,62_ = 52.56, *p* < .0001), and leucine (*F*_1,62_ = 33.88, *p* < .0001, Figure 5).

## 4 DISCUSSION

We report differential resource use between sexes of a rare butterfly across a 21‐year period. Ours joins a handful of other studies that have examined sex‐specific foraging in nectar‐feeding species (reviewed by Smith et al., 2019). Our study is distinct because of its long‐term perspective; most studies have typically been conducted on the order of a single season and therefore cannot capture interannual variation. We also take preliminary steps to identify potential mechanisms driving divergent foraging behavior, which has only been studied in a limited capacity. Our work suggests that differences in foraging patterns between male and female *A. i. idalia* are partially driven by phenology, but also that other mechanisms likely influence these patterns, given sex‐specific floral visitation often remains robust during periods of male–female overlap. Nutritional differences in nectar may play a role, as females were more likely to nectar on plants with higher concentrations of total carbohydrates as well as specific individual sugars and AAs.

### 4.1 Trends in sex‐specific foraging patterns in *A. i. idalia* across two decades

Our results reinforce findings that the average difference between foraging patterns of male and female floral visitors within species can be as great or even greater than average interspecific differences (Smith et al., 2019). Our estimation of mean dissimilarity between male and female *A. i. idalia* foraging across years based on the Morisita–Horn dissimilarity index (0.37 *d*_mh_) is at least on par with the average found in a study of sex‐specific foraging of 19 bee species within a year (~0.40 *d*_mh_, Roswell et al., 2019), the only other study using the same metric for sex‐specific floral foraging. We also found a 0.77 difference in effective species number between female and male *A. i. idalia*, which, although seems small, nevertheless represents a ~30% increase in effective species number for females relative to males. Similarly low numbers of nectar plants per year are observed in other butterfly species, such as the Adonis blue butterfly (3–8 species), which also exhibits greater floral resource breadth in females than males (Rusterholz & Erhardt, 2000). The difference in nectar plant diversity between male and female *A. i. idalia* is also greater than the average male–female difference reported for other flower‐foraging species by Smith et al. (2021). They estimated that females visit 0.5 more plant species than males, and notably their estimate is based on species richness which in contrast to effective species number does not account for evenness of visitation to plant species. In sum, the differences in foraging patterns that we reveal for *A. i. idalia* seem to reflect biological significance.

In observing *A. i. idalia* nectaring events for over two decades, we were also able to capture interannual *variation* in sex‐specific foraging patterns. We found that although females and males consistently diverged, there was substantial interannual variation in how much they diverged. In fact, the range of *d*_mh_ dissimilarity index values across years for *A. i. idalia* (0.01–0.89, considering the entire season) rivals the interspecific variation in mean male–female dissimilarity reported for bee species (0.1–0.8, Roswell et al., 2019). It is possible that small sample sizes in a few years due to temporal variation in *A. i. idalia* population size (Zografou et al., 2017) may simply have reduced the accuracy of our measurement of sex‐specific differences. However, given the strong effect of phenology on sex‐specific foraging patterns in *A. i. idalia*, we posit that at least some of the interannual variation in the degree of divergence between males and females is a product of interannual variation in the timing of sex‐specific life history schedules (Figure 1a), which we discuss below. Because studies are often limited in their time scale, we do not know how common such variation in sex‐specific foraging is in other species, despite demonstrable interannual variation in the amount of generalization in pollinator communities more broadly (e.g., Alarcón et al., 2008; Petanidou et al., 2008). Regardless, our finding of large variation in male–female differences in foraging patterns across year suggests that diet flexibility (Morán‐López et al., 2022) can play in modulating sex‐divergent foraging.

### 4.2 Pointing toward mechanisms underlying sex‐specific foraging patterns in *A. i. idalia*

Our data on *A. i. idalia* foraging indicate a large role of sex‐specific phenology in driving divergent foraging patterns across a season, mirroring the role that interspecific differences in phenology have been shown to play in determining plant–pollinator species associations in entire communities (CaraDonna et al., 2017; Encinas‐Viso et al., 2012; Morente‐lópez et al., 2018; Ogilvie & Forrest, 2017; Roswell et al., 2019). First, the difference between the diversity of species visited by males and females disappears when exclusively considering the period of overlap. Second, accounting for phenology in this way erased or even reversed a signal of dissimilarity in eight of the years that regals were observed. In the remaining years, foraging differences were still greater than expected during the overlap period but often substantially lower than those found for the entire season. Roswell et al. (2019) similarly found an impact of phenology on sex‐specific foraging patterns of several bee species. Though they estimated a weak effect, it is possible that the relative impact of phenology on these patterns is taxon‐specific; reward offering might have a relatively greater influence on bees, since male bees only visit nectar‐producing species but female forage for pollen and nectar. Nevertheless, given the prevalence of protandry in bee and butterfly species (Bourke, 1997; Wiklund & Fagerström, 1977) differences in phenology between sexes such as those found in the *A. i. idalia* are likely to generate intraspecific variation in foraging in other species as well.

Explicit consideration of the interaction between regal and flowering plant phenology at our study site illustrates how the difference in the diversity and identity of nectar plant associations can arise between male and female *A. i. idalia*. Community‐wide floral diversity at one of the grasslands in our study was found to be lower at the start of the season, increasing, and then plateauing after the first 2 weeks, at least based on a single season (G. X. Smith, unpublished data). With respect to *A. i. idalia*, this means that males are emerging when diversity is at its lowest, whereas females emerge later when diversity is greater. Since diversity in seasonal environments often exhibits a “phenological mid‐domain” effect, with diversity initially low, increasing toward a peak, and declining at the end of the season (Morales et al., 2005), greater diversity of female‐associated plants might be expected in protandrous pollinators more generally. Moreover, *A. i. idalia* females remain on the landscape for a greater time, so they cumulatively encounter a greater diversity of nectar species as flowering plant species turn over across the season. With respect to individual plant species, *Asclepias* spp. are among the earliest plants to flower during the period of regal activity and commonly among the first nectaring events recorded each season. In fact, peak flowering of the two male‐associated *Aslcepias* aligns with *A. i. idalia* male emergence and activity on the landscape, while their flowering ends or at least strongly declines prior to peak female abundance. Likewise, female‐associated *C. discolor* blooms late in the season when males are declining but females are still active. In essence, certain sex–plant associations become akin to “forbidden links” (Olesen et al., 2011). Szigeti et al. (2019) similarly made this connection at the level of individual *Parnassius mnemosyne* butterflies. By tracking visitation of individual butterflies across their lifetime and shifts in floral abundance across a growing season, they demonstrated that apparent individual specialization was linked to changes in relative abundance of floral resources. Indeed, this and another study emphasize that shifts in relative abundance of flowers on the landscape throughout the season, in addition to floral species turnover, influence floral resource use (Szigeti et al., 2018, 2019). In this way, one can also envision why there is so much year‐to‐year variation in dissimilarity in male and female *A. i. idalia* foraging patterns. A change in dissimilarity only requires plants to shift in timing of flowering or shifting dates of male versus female pollinator emergence, peak abundance, or death (Figure 1b), yet combinations of these are likely to occur, highlighting the importance of long‐term studies. In fact, post hoc analysis of our data indicates that 30% of the year‐to‐year variation in foraging dissimilarity in *A. i. idalia* across an entire season can be explained by variation in the time between median dates of male and female *A. i. idalia* foraging; the farther apart these dates, the greater the dissimilarity ($R_{\mathrm{adj}}^{2}$=0.30, *p* = .006, data not shown). Following floral abundances across and within years can help test this hypothesis (e.g., Szigeti et al., 2018).

Still, remaining signals of sex‐specific foraging patterns after accounting for phenology underscore a role for other mechanisms. First, the six most visited species in our longitudinal study make up only a small proportion of the available floral units compared to their frequency of visitation; in one year it was estimated they represented only 3.3% of flowering plants in one of the grasslands (G. X. Smith, unpublished data). This suggests preference and not just simply abundance plays a role in determining visitation patterns. Our nectar chemistry results also point toward associations between nectar nutrition and floral visitation patterns. Plants more commonly visited by female *A. i. idalia* had greater nectar concentrations of leucine, glycine, and proline, which aligns with previous demonstrations of female preference and male indifference or aversion toward AA‐rich nectar in several lepidopteran species (Alm et al., 1990; Erhardt, 1992; Mevi‐Schütz & Erhardt, 2002, 2003). This is consistent with the possibility that nectar AA may be allocated toward egg production and larval development (Levin et al., 2017a, 2017b). Nectar sugar can also be important for egg production—upwards of 80% of egg carbon was gained from sugar in the congener *Argynnis mormonia* (O'Brien et al., 2004)—and nectar sugar has been shown to be used in de novo non‐essential AA production (Brien et al., 2005). This could perhaps account for the lack of difference in total nectar AA concentrations but greater total carbohydrates in nectar of female‐associated species. With respect to specific sugars, greater glucose and fructose concentrations in female‐associated nectars are in accord with female preference for glucose in *L. bellargus* (Rusterholz & Erhardt, 2000) and high sensitivity to fructose in *Pieris brassicae* (Romeis & Wäckers, 2000), but similar sucrose levels do not follow findings of male‐preference in other species (Alarcón et al., 2010; Rusterholz & Erhardt, 2000). Greater individual AA and total and component sugar concentrations in female‐associated species at first seem to suggest there is no tradeoff and males rely on poorer quality nectar. Notably, we tested only a select subset of AAs based on a priori hypotheses, none of which are correlated with total AA content (*p* > .15, *N* = 68, data not shown), leaving the possibility that other AAs could be more important to males. However, males may also have fewer nutritional constraints. They may spend more time feeding on flowers with more abundant nectar that is less concentrated, substituting quality for quantity. Finally, male foraging patterns may reflect a role of nutrients other than those our work focuses on, such as sodium, or alternative strategies, leading them to supplement their diet with alternative sources such as mud puddles. For example, males of congener *A. mormonia* feed at mud puddles to collect sodium, whereas females do not (Boggs & Jackson, 1991), and we have seen mud‐puddling by *A. i. idalia* at Fort Indiantown Gap (M. T. Swartz, personal observation).

There are limitations to our nectar data. Most notably, we could not evaluate all plant species. For the six we examined, males were associated more strongly with both *Asclepias* species and three of the female species are in the Asteraceae family¸ potentially confounding phylogeny with ecology. However, several nectar compounds differed between the *Asclepias* species and among the Asteraceae species, suggesting nectar chemistry is not strongly conserved (Figure 4, Table S1). Additionally, while comparative nectar studies routinely use a per volume basis as a comparison as we have done, nectar volume may be an important component. Other floral traits may be as or more important and similarly confounding. Certainly, color can influence visitor identity at the species level (Bradshaw & Schemske, 2003), and the female‐associated species we examined here are all purple, though so is *Asclepias syriaca*. Regardless, our inferences regarding nectar chemistry provide an intriguing initial accounting of an association between nectar constituents and sex‐specific differences in floral visitation. Future work including nectar volume and chemistry of all species visited by *A. i. idalia* combined with controlled preference studies (Erhardt, 1992; Mevi‐Schütz & Erhardt, 2003, 2004) can directly address whether the associations we report here persist and are due to active foraging choices rather than, for example, developing as a consequence of phenological differences in males and females layered atop a changing nectar landscape across the season (Nottebrock et al., 2016).

## 5 CONCLUSIONS AND IMPLICATIONS

The patterns revealed in our study have both broad ecological consequences and potential conservation implications. Our findings provide a link between work on how sex‐specific foraging patterns can shape network interaction topologies (Kishi & Kakutani, 2020; Smith et al., 2021) and work emphasizing temporal variation in plant–pollinator networks (CaraDonna et al., 2017; Mora et al., 2020; Olesen et al., 2008; Schwarz et al., 2020). Indeed, our work highlights how divergent emergence time and thus foraging periods between male and female pollinators can create forbidden links that would otherwise be ignored and provides new insight into a potential mechanism driving interaction rewiring (CaraDonna et al., 2017). For instance, rewiring could be attributable to the combination of sex‐specific foraging patterns and changing ratios of males to females across the season.

Our findings also raise the possibility that restoration programs targeting rare or threatened species like *A. i. idalia* could benefit from considerations of sex differences in foraging. For example, conservation of the *A. i. idalia* population involves anthropogenic maintenance of the flowering community and habitat restoration projects. Accounting for sex‐specific differences could enable projects to target planting the most visited species while simultaneously ensuring that male‐ and female‐specific requirements are met. Additionally, given that intersexual differences in floral visitation are partially driven by phenology, it is possible that plant–pollinator mismatches caused by anthropogenic climate change could exacerbate male–female differences or differentially impact the sexes. For example, *Asclepias syriaca* has already been shown to flower earlier with increased temperature due to anthropogenically driven climate change (Howard, 2018). Although some studies suggest that phenological tracking may be sufficient to avoid phenological mismatch in some plant–pollinator mutualisms in response to climate change (Forrest, 2015; Forrest et al., 2010; Theobald et al., 2016, 2017), others show mismatches, especially early in the season (Kudo & Ida, 2013; McKinney et al., 2012). In protandrous pollinators, males may therefore be particularly susceptible to such climate‐driven mismatches, which could in turn reduce numbers of males available for mating and diminish the genetic diversity of subsequent generations. A similar climate‐driven mismatch in protandrous species could also occur late in the season for females, with even more direct consequences for reproduction and population growth. Conservation practitioners might therefore focus on maintaining or expanding the provision of nectar throughout the extended period of adult foraging that is expected for some species (such as *A. i. idalia*; Zografou et al., 2021) under climate change.

## AUTHOR CONTRIBUTIONS

**Matthew W. Chmielewski:** Data curation (supporting); formal analysis (lead); methodology (equal); software (lead); validation (equal); visualization (lead); writing – original draft (lead); writing – review and editing (lead). **Skyler Naya:** Formal analysis (supporting); writing – original draft (supporting); writing – review and editing (supporting). **Monica Borghi:** Investigation (lead); methodology (equal); validation (equal); writing – review and editing (supporting). **Jen Cortese:** Investigation (supporting); writing – review and editing (supporting). **Alisdair R. Fernie:** Investigation (supporting); methodology (equal); resources (equal); writing – review and editing (supporting). **Mark T. Swartz:** Conceptualization (supporting); data curation (equal); investigation (lead); methodology (equal); project administration (supporting); resources (equal); supervision (supporting); validation (equal); writing – review and editing (supporting). **Konstantina Zografou:** Data curation (equal); validation (equal); writing – review and editing (supporting). **Brent J. Sewall:** Data curation (equal); funding acquisition (equal); validation (equal); writing – review and editing (supporting). **Rachel B. Spigler:** Conceptualization (lead); funding acquisition (equal); methodology (equal); project administration (lead); resources (equal); supervision (lead); writing – original draft (supporting); writing – review and editing (lead).

## FUNDING INFORMATION

This research was supported by funding from the Pennsylvania Department of Military and Veterans Affairs (4300420055 and 4300599299) to RBS and BJS.

## CONFLICT OF INTEREST STATEMENT

The authors declare no conflicts of interest.

## Supporting information


Supporting Information and Figures S1–S5

Table S1

## Data Availability

All data and code will be made publicly available via Dryad upon publication and are currently available at https://github.com/mwchmiel/sexspecificspeyeriaFIG for review.
