# Supplementary material for: Phenology and foraging bias contribute to sex‐specific foraging patterns in the rare declining butterfly Argynnis idalia idalia
Source: Ecol Evol. 2023 Jul 18;13(7):e10287. doi: 10.1002/ece3.10287 (PMC10353922; doi:10.1002/ece3.10287)
Supplement: Supplementary file 1 — Supporting Information and Figures S1–S5 [file ECE3-13-e10287-s001.docx]

| **Supporting Information & Figures**  **Supporting Methods**  **Supporting Figures S1-5** |
| --- |
| **Article Title**: Phenology and nectar chemistry contribute to sex-specific foraging patterns in the rare declining butterfly *Arynnis idalia idalia* |
| **Authors:** Matthew W. Chmielewski, Skyler Naya, Monica Borghi, Jen Cortese, Alisdair R. Fernie, Mark T. Swartz, Konstantina Zografou, Brent J. Sewall, Rachel B. Spigler |

**Supporting Methods:** On assignation of *Cirsium spp.* accounts

We note that in some cases two thistle species (*Cirsium pumilum* and *Cirsium discolor,* Asteraceae), were recorded only as ‘Cirsium spp.’, comprising 29% of all *Cirsium* accounts. These species exhibit strongly distinct temporal flowering distributions at our site, with *C. pumilum* flowering earlier than *C. discolor*, though there is a small amount of overlap in the late and early tails of their distributions, respectively. Analyses pooling all *Cirsium* observations would therefore strongly obscure any potential differences in sex-specific patterns of flower visitation, particularly those associated with phenological differences between male and female butterflies. To address this, we assigned unknown *Cirsium* individuals to either *C. pumilum* or *C. discolo*r based on the empirical distributions of flowering dates of each species (when explicitly classified) via a bound-constraint support vector machine (SVM) learning approach using the kernlab package for R (Karatzoglou et al. 2004, 2006). When applied to unknown *Cirsium* visitations, the model classified all but 30 observations to either *C. pumilum* or *C. discolor*. Of the remaining 30 ambiguous accounts, 24 were late season observations, occurring after the latest recorded date of *C. pumilum* visitation in our dataset. Thus, these observations could not be *C. pumilum* and must represent the long tail end of *C. discolor*’s flowering distribution. We manually reclassified these 24 late season observations as *C. discolor* and dropped the remaining ambiguous 6 observations from the dataset. To test the performance of our classification model, we applied it to our known species accounts; it properly reclassified *C. pumilum* and *C. discolor* observations to 96% and 92% accuracy, respectively, with the remaining accounts reclassified as unknown *Cirsium spp.*, and no reclassifications from one known species to the other, supporting the application of our approach. Finally, as *Centaurea* species were difficult to disambiguate in the field and have similar flowering periods, members of this genus were lumped as *Centaurea spp.* for all subsequent analyses.

Karatzoglou, A., D. Meyer, and K. Hornik. 2006. Support Vector Machines in R. Journal of Statistical Software 15:1–28.

Karatzoglou, A., A. Smola, K. Hornik, and A. Zeileis. 2004. kernlab -- An {S4} Package for Kernel Methods in {R}. Journal of Statistical Software 11:1–20.


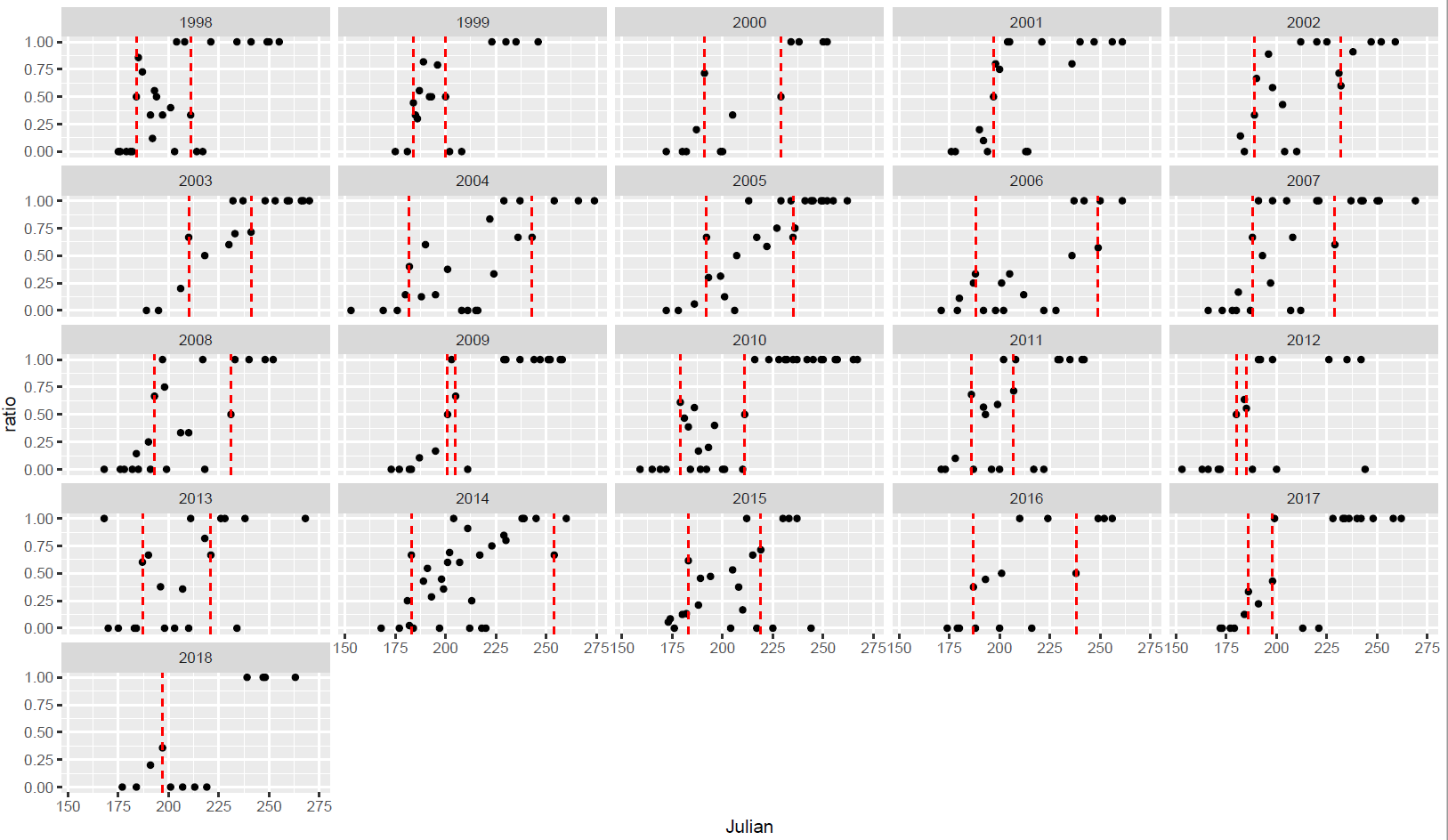
**Figure S1:** Sex ratio of *Argynnis idalia idalia* observations by year used to construct a phenological overlap dataset. Periods depicted between vertical red lines are inclusive of the first and last dates in which the female:male sex ratio was between 0.25 and 0.75, and thus define a conservative overlap dataset. Given these criteria, 2001 and 2018 are reduced to single observation days, and were removed from subsequent analyses on overlap data.


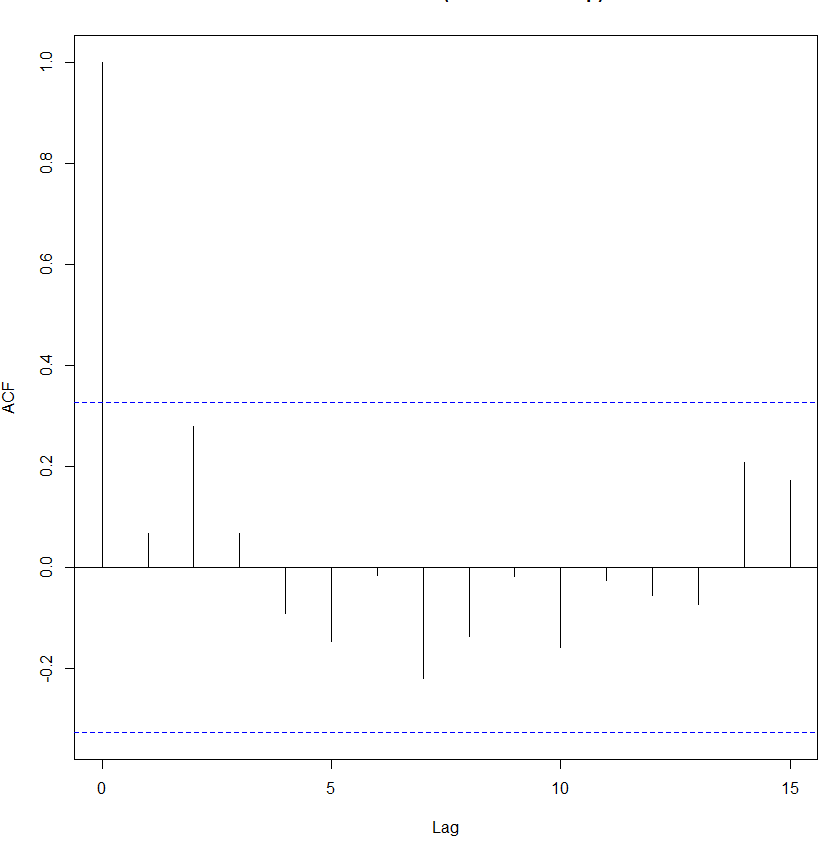

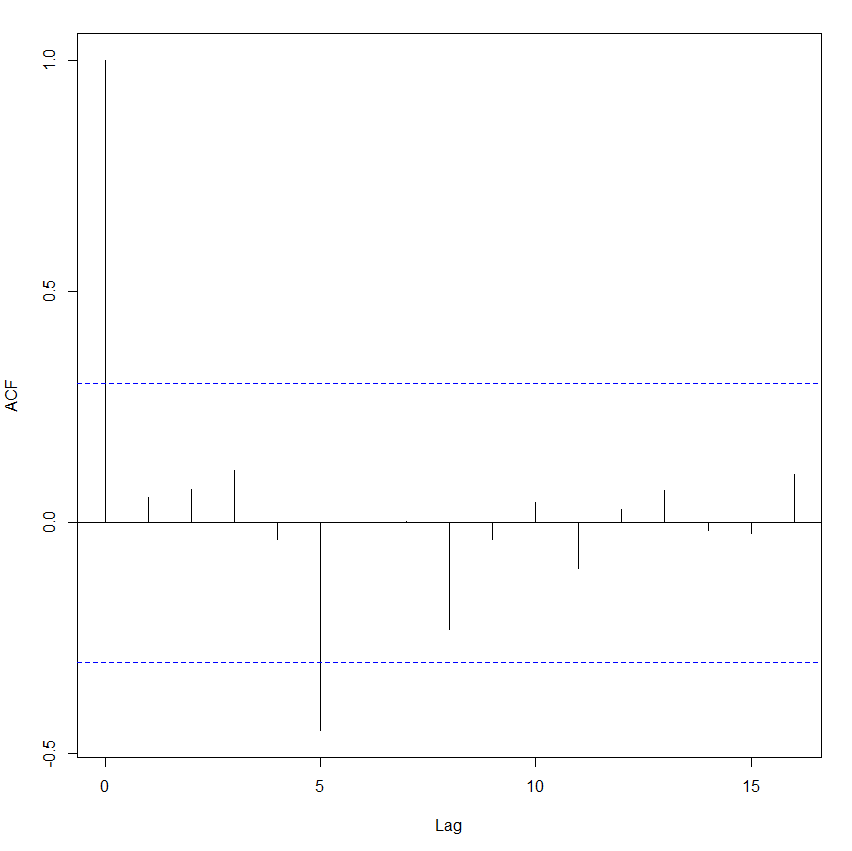


**Figure S2:** Lag structure of effective species number residuals (left), indicating a significant lag 5 structure. When a single year (2009) was removed, no significant lag structure was detected (right), suggesting a lack of consistent autocorrelation in effective species number across our 21-year dataset.


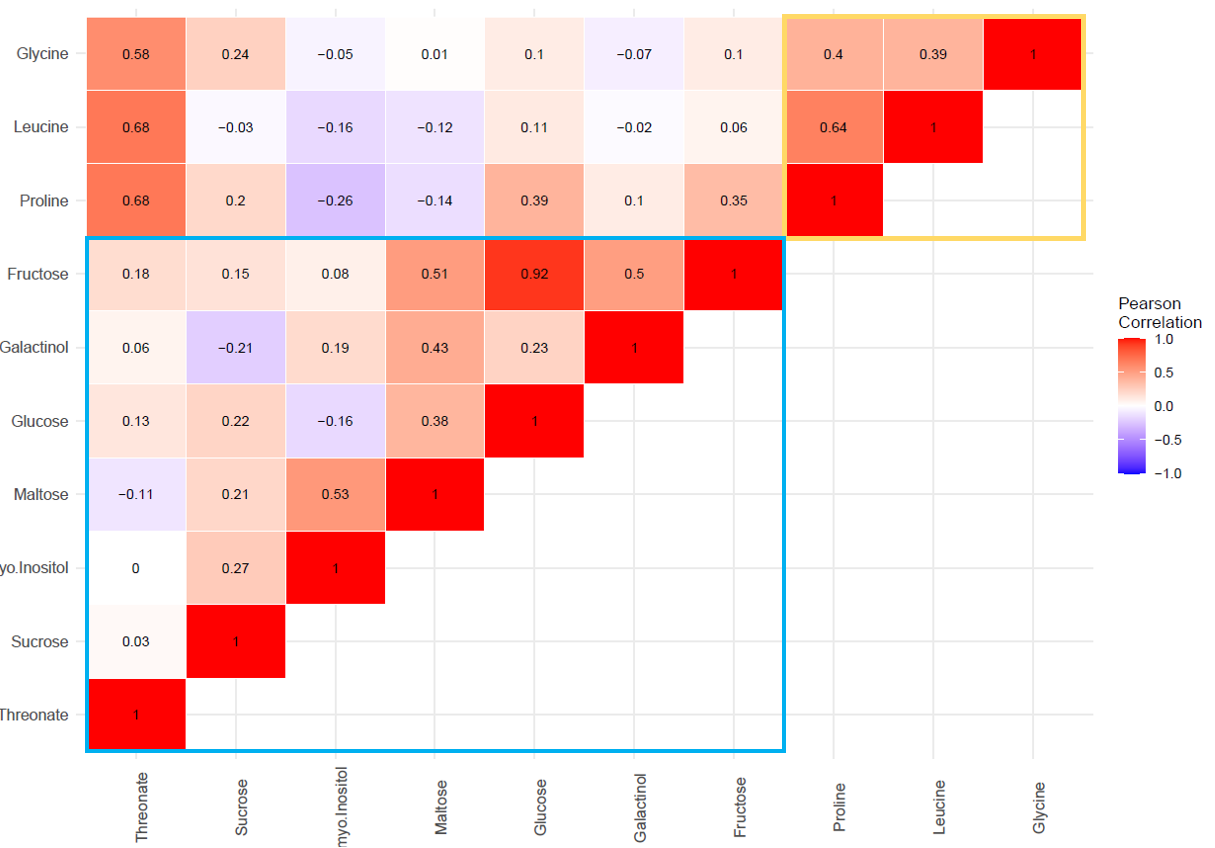


**Figure S3:**  Pearson correlation coefficients between individual compounds found in nectar from six sampled flower species. Carbohydrates are denoted by the blue box, while amino acids are denoted by the gold box. Correlation coefficients range from 0-1, with 1 being perfect correlation between compounds. Compounds that are positively correlated are in red, while those that are inversely correlated are in blue, with color density indicated degree of correlation.


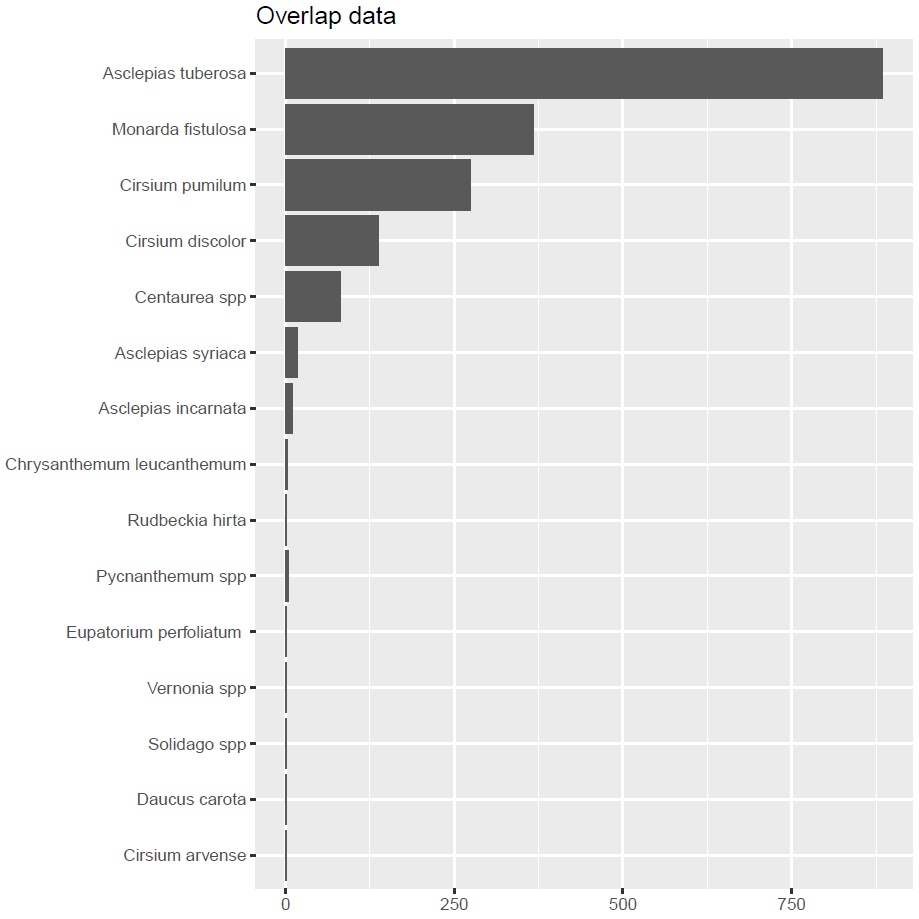

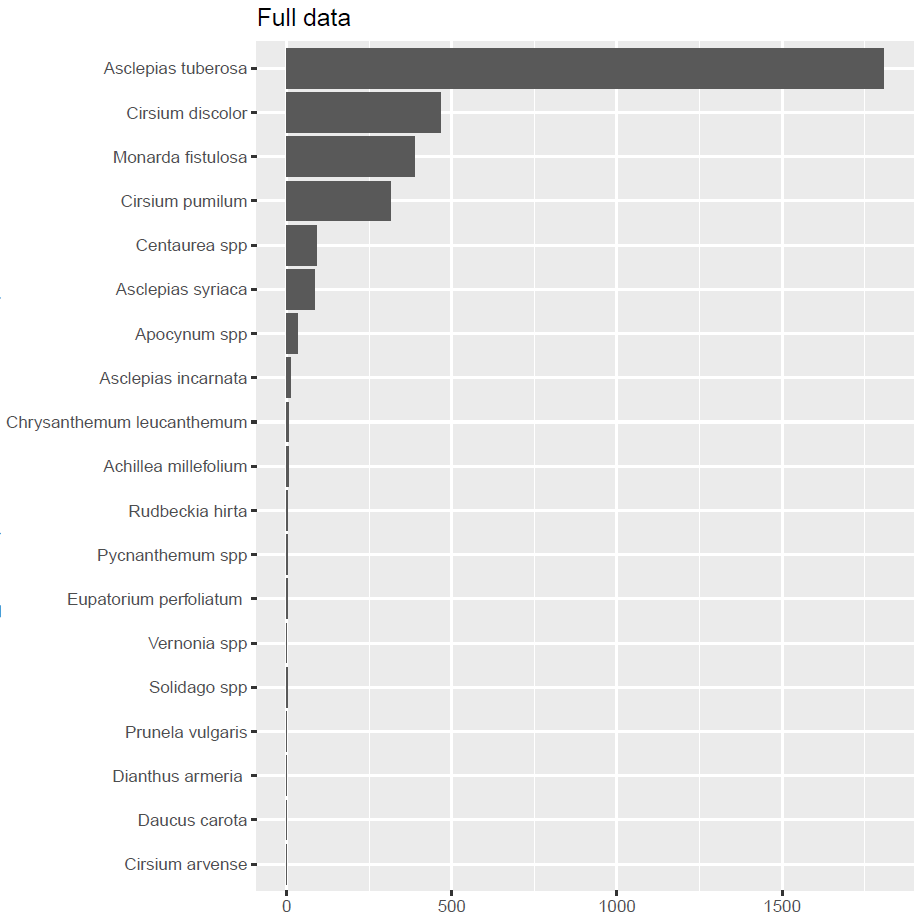


**Figure S4:** Plant species ranked by most *Argynnis idalia idalia* visitations in the full (left) and overlap (right) datasets.


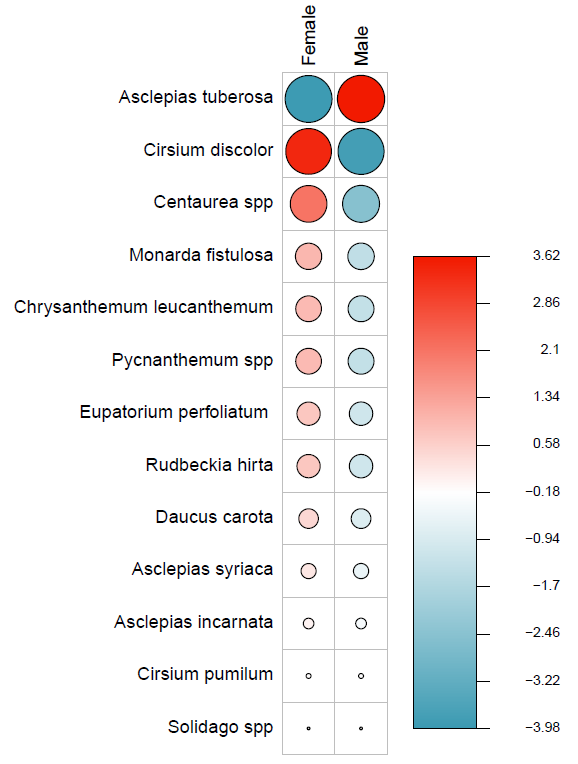


**Figure S5:** Sex-specific associations with plant species visited by eastern regal fritillaries constrained by a core period of male and female overlap in activity, across all years. The size of circles and color saturation (scale bars) indicate larger residuals and thus a greater association, with red denoting visitation is greater than that expected by abundance alone (i.e., positive association) and blue denoting negative association. Female and male preferences in these years are similar to preferences we found in the overall dataset.
